# The Role of Gut Microbiota in the Etiopathogenesis of Multiple Chronic Diseases

**DOI:** 10.3390/antibiotics13050392

**Published:** 2024-04-25

**Authors:** Lara Pires, Ana M. González-Paramás, Sandrina A. Heleno, Ricardo C. Calhelha

**Affiliations:** 1 Centro de Investigação de Montanha (CIMO), Instituto Politécnico de Bragança, Campus de Santa Apolónia, 5300-253 Bragança, Portugal; laravaqueiro@ipb.pt (L.P.); sheleno@ipb.pt (S.A.H.); 2 Grupo de Investigación en Polifenoles en Alimentos, Implicaciones en la Calidad y en Salud Humana, Facultad de Farmacia, Universidad de Salamanca, Campus Miguel de Unamuno s/n, 37007 Salamanca, Spain; paramas@usal.es; 3 Laboratório Associado para Sustentabilidade e Tecnologia em Regiões de Montanha (SusTEC), Instituto Politécnico de Bragança, Campus de Santa Apolónia, 5300-253 Bragança, Portugal

**Keywords:** chronic diseases, gut microbiota, dysbiosis

## Abstract

Chronic diseases (CD) may result from a combination of genetic factors, lifestyle and social behaviours, healthcare system influences, community factors, and environmental determinants of health. These risk factors frequently coexist and interact with one another. Ongoing research and a focus on personalized interventions are pivotal strategies for preventing and managing chronic disease outcomes. A wealth of literature suggests the potential involvement of gut microbiota in influencing host metabolism, thereby impacting various risk factors associated with chronic diseases. Dysbiosis, the perturbation of the composition and activity of the gut microbiota, is crucial in the etiopathogenesis of multiple CD. Recent studies indicate that specific microorganism-derived metabolites, including trimethylamine N-oxide, lipopolysaccharide and uremic toxins, contribute to subclinical inflammatory processes implicated in CD. Various factors, including diet, lifestyle, and medications, can alter the taxonomic species or abundance of gut microbiota. Researchers are currently dedicating efforts to understanding how the natural progression of microbiome development in humans affects health outcomes. Simultaneously, there is a focus on enhancing the understanding of microbiome–host molecular interactions. These endeavours ultimately aim to devise practical approaches for rehabilitating dysregulated human microbial ecosystems, intending to restore health and prevent diseases. This review investigates how the gut microbiome contributes to CD and explains ways to modulate it for managing or preventing chronic conditions.

## 1. Introduction

In a world marked by scientific innovations promising longevity and well-being, it is possible to increasingly witness not only a rise in Chronic Diseases (CD), responsible for about 60% of worldwide deaths [1], but also a decline in the quality of life, compromising the potential for active ageing. Experts estimate that by 2025, at least one in four individuals may have one CD [2].

In conformity with the CDC (Centers for Disease Control and Prevention), a Chronic Disease is considered a condition that lasts for more than a year, requiring ongoing medical attention and limitations in daily activities [3]. Genetics, lifestyle, social behaviours, healthcare systems, community influence, and environmental factors influence these diseases. Modern civilizations face a progressive increase in immune-mediated or inflammatory health problems, such as autoimmune disorders, allergic diseases, obesity, and metabolic diseases [4]. Given their lengthy duration and gradual advancement, CDs inflict a significant financial strain on healthcare systems financially and structurally [5].

Many studies point to population ageing as a strong driver of CD. Additionally, the globalization of unhealthy lifestyles [6], unbalanced diets with high levels of processed food, and low consumption of fibre, vitamins, and minerals contribute significantly to the increased risk of CD [7]. Adverse outcomes related to these pathologies occur on a scale and intensity not anticipated by different health organizations [8]. CD hinders each country’s national economic development, resulting in productivity losses due to the inability or limitation of daily work [9], which the pathology restricts, affecting the quality of life and increasing medical needs and healthcare expenses. This public health issue emphasizes the importance of CD prevention and early diagnosis [10].

The intestines are home to a diverse array of microbial species. Extensive research has delved into the entirety of this microbial population and their genetic makeup (microbiome) [8] using advanced methodologies such as metagenomics, metatranscriptomics, and metabolomic analyses. These studies have revealed that disruptions in the microbial community can upset intestinal equilibrium [11], leading to cascading effects on various bodily systems. Importantly, these investigations underscore the pivotal role of adjustments in the configuration and efficacy of the gut microbiota in the onset and complications of cardiovascular diseases (CVD) [12], type 2 diabetes [13], and chronic kidney disease (CKD) [14].

This review delves into the intricate ways in which the gut microbiota (GM) and its metabolites impact cellular targets downstream, contributing to the development of CVD, type 2 diabetes, and CKD. Importantly, it also explores the potential therapeutic implications that could arise from these findings, offering a promising direction for future research and treatment.

## 2. Gut Microbiota: Its Complexity and Dynamics

Dating back to Hippocrates (400 B.C.), the influence of the intestine on human health has been recognized, with the famous saying, “Death sits in the intestine” [15]. The microbiome is synonymous with a genomic collective of microorganisms inhabiting an environmental niche [16,17]. Microbiota is the collection of microorganisms forming an ecological community of symbiotic, commensal, and pathogenic microorganisms that share our body space [18].

The colonization of the gastrointestinal tract begins before birth, through the mother via the placenta. Experts estimate that our bodies harbour over 100 trillion bacterial cells (mostly non-pathogenic bacteria) [16] throughout our lives, comprising 1.05 to 2 kg of our weight [19]. The intestinal microbiota becomes an increasingly dynamic and diverse ecosystem after birth, influenced by factors such as diet (breast milk or formula), delivery method (vaginal or c-section), infant hygiene, and antibiotic use [20]. Stabilization and similarity with the adult microbiota occur around 2–3 years [21]. For healthy individuals, the arrangement of the intestinal microbiota remains relatively stable and similar among individuals from the same region or with a similar diet.

Despite its vigorous metabolic activity, the intestinal microbiota possesses a remarkable capacity to acclimate to changes in the intestinal environment [22]. It adjusts to the type of nutrients available, leading to modifications and alterations in the enzymes produced. Although not fully explained, this adaptability is evident in faecal bacteria such as *E. coli*, which divide every 20 min, demonstrating their genetic adaptability to the environment [23]. Without such adaptability, humans would have been unable to handle changes in lifestyle and dietary habits, as demonstrated by the shift from the Palaeolithic era to the dietary practices of modern societies.

The human microbiome consists of more than 3 million genes and is the subject of investigation by the Human Microbiome Project Consortium [24]. These studies have identified the gut microbiota primarily by five bacterial phyla: *Firmicutes*, *Bacteroidetes*, *Actinobacteria*, *Proteobacteria*, and *Verrucomicrobia* [22]. The most common species in the human intestine (90% of all species) include *Firmicutes* (*Ruminococcus*, *Clostridium*, *Lactobacillus*, *Eubacterium*, *Faccalibacterium*, and *Rosaburia*) and *Bacteroidetes* (*Bacteroides*, *Provetella*, and *Xylanibacter)* (Table 1). Most bacteria have anaerobic metabolism, leading to reduced oxygen tension [25]. These microorganisms inhabit different ecological niches on mucosal surfaces in the gut lumen, with their density increasing along the intestine, forming a complex biochemical interaction network between the host and the bacteria [26].

The mutualistic association between the intestinal microbiota and the host is crucial for maintaining health. The intestinal microbiota is vital in regulating barrier functions, immune stimulation, trophic functions, metabolism, and signalling to virtually all body organs [1]. Metabolites of the intestinal microbiota, such as lipopolysaccharides (LPS) and peptidoglycan, can directly interact with host cells via toll-like receptors (TLRs) [32].

The recent breakthroughs in understanding the intricate structure and function of microbial communities have sparked a new era in microbiome research. The once-underestimated gut microbiome is now acknowledged as a significant player in host physiology, metabolism, and disease [33]. However, its role in pharmacology remains largely uncharted territory. For almost a century, it has been known that human gut microbes can metabolize drugs, such as the activation of the antibiotic prontosil by gut bacteria, which influences its antibacterial activity. This biotransformation extends to various compounds, including azo dyes and drugs like sulfasalazine, used to treat ulcerative colitis and rheumatoid arthritis [34]. The extent of drug biotransformation by the gut microbiota is far more extensive than previously believed. Gut microbes exert their influence on drug therapy through direct and indirect mechanisms. Direct mechanisms involve modifying drugs or their metabolites into products with altered bioactivities. Indirect mechanisms encompass intricate host–microbial interactions that affect xenobiotic metabolism or transport [35]. The interconnection between drugs and the gut microbiome is bidirectional, impacting both the drug’s efficacy and potential side effects. When orally administered drugs enter the body, they often encounter the gut microbiota before reaching the liver or kidneys. Notably, drug coatings do not prevent these microbial interactions [36]. A significant study revealed that 66% of 271 non-antibiotic drugs were metabolized by at least one of 76 gut bacterial species [37].

Drugs can exert inhibitory or beneficial effects on bacteria within the gut, either globally or selectively. Concurrently, gut microbes can metabolize drugs to produce active, inactive, or toxic compounds [36]. Drug metabolism is primarily carried out by the liver through various reactions such as conjugation, hydrolysis, oxidation, and reduction. These processes yield Bile Acids (BAs), which interact with the gut microbiome and are essential communication substrates between the liver and gut [38].

For instance, metformin promotes the expression of genes in bacteria that respond to environmental changes, leading to increased production of BAs and Short-Chain Fatty Acids (SCFAs) [39]. Statins, conversely, may interact significantly with BAs produced by gut microbes. Levels of primary and secondary BAs have been shown to influence the effectiveness of simvastatin in lowering LDL cholesterol [40].

While antibiotics are the most evident drugs affecting gut microbes, leading to reduced SCFAs and BAs and increased susceptibility to *Clostridium difficile* infections [41], non-antibiotic medications also have direct effects. In vitro studies revealed that 24% of non-antibiotic compounds curtailed the growth of at least one bacterial strain, with antipsychotics and proton pump inhibitors (PPIs) showing the most potent inhibitory effects [42]. PPIs were found to have lasting impacts on the gut microbiota, associated with adverse health outcomes even after discontinuation. Additionally, drugs like simvastatin, gemcitabine, digoxin, and L-dopa are metabolized by gut bacterial enzymes, influencing their activation or inactivation [43]. Interestingly, the gut microbiome can also contribute to drug–drug interactions. For instance, simvastatin can enhance the efficacy of gemcitabine by reducing its resistance through inflammatory pathways modulated by the microbiome [44].

Despite the structural diversity of drugs metabolized by the gut microbiota, common mechanisms such as reduction and hydrolysis are prevalent, likely reflecting the microbes’ energy needs [36]. The microbiome also facilitates reactions like acetylation and deacetylation, impacting the metabolism of drugs like acetaminophen and levodopa [43].

The significance of the gut microbiota stretches beyond pharmacology to nutrition. Altered drug metabolism by gut microbes can lead to dysbiosis, potentially worsening chronic diseases that require long-term drug treatments. Metabolites from these drugs can further alter the gut flora, contributing to dysbiosis or producing microbial metabolites that may worsen or trigger specific pathologies [38]. The growing recognition of the gut microbiota’s role in pharmacology and nutrition unveils the transformative potential of this research. Advancements in this field could pave the way for strategies to enhance drug outcomes by manipulating the gut microbiota or predicting drug responses through metabolite or genetic screening [45].

The gut microbiota’s influence extends beyond pharmacology to nutrition. Altered drug metabolism by gut microbes can lead to dysbiosis, potentially exacerbating chronic diseases requiring long-term drug treatments. Metabolites from these drugs can further alter the gut flora, contributing to dysbiosis or producing microbial metabolites that may exacerbate or trigger specific pathologies. The growing recognition of the gut microbiota’s role in pharmacology and nutrition reveals the translational potential of research. Advancements in this field could lead to strategies to improve drug outcomes by manipulating the gut microbiota or predicting drug responses through metabolite or genetic screening [45].

Among its various physiological functions, the gut microbiota’s involvement in the food digestion process stands out. For instance, complex polysaccharides, initially digested by intestinal enzymes in the small intestine, are further metabolized by the microflora [38]. The presence of undigested carbohydrates (CHO) and proteins in the intestinal lumen promotes the metabolism of anaerobic bacteria. However, the final products of these bacteria’s fermentation can vary widely, leading to different effects on humans. These polysaccharides (CHO) are degraded and fermented, converting into Short-Chain Fatty Acids (SCFAs) and gases (methane and hydrogen) [46]. SCFAs lower intestinal pH, constrain the multiplication of pathogenic bacteria and function as a source of energy for intestinal cells, contributing to energy expenditure, glucose homeostasis, and satiety. The primary short-chain fatty acids (SCFA) of significance include butyrate, a crucial energy source for colonic epithelial cells, along with acetate and propionate, which serve as substrates for lipogenesis and gluconeogenesis [47]. These compounds also play a role in positively modulating insulin secretion [11]. In the presence of sufficient undigested carbohydrates (i.e., dietary fibres), proteins are primarily utilized for bacterial growth, promoting saccharolytic bacterial species such as *Bifidobacterium* and *Lactobacillus*, which predominantly ferment carbohydrates [11].

Under conditions of reduced carbohydrate availability, proteins undergo increased fermentation by proteolytic bacteria, with *Clostridium* and *Bacteroides* being the main proteolytic species. This process generates energy through deamination. However, it is crucial to note that protein fermentation pathways also produce potentially harmful metabolites (ammonia, amines, thiols, phenols, and indoles) [48]. Typically, these metabolites are excreted in faeces and eliminated by the kidneys. The regulation of bacterial metabolism is heavily influenced by the availability and composition of nutrients, especially the balance between undigested carbohydrates (CHO) and protein. Due to the efficient fermentation of carbohydrates by bacteria, the CHO/protein ratio decreases along the length of the intestine. However, a slowed transit, such as constipation, can lead to an overgrowth of proteolytic species, promoting the production of both toxic metabolites and pro-inflammatory substances [48].

The intestinal microbiota is also an endogenous synthesis of specific vitamins and amino acids. Metabolic regulation in the intestine or distal organs through microbial metabolites, including bile acids, SCFAs, Trimethylamine-N-oxide (TMAO), peptide YY, and glucagon-like peptide 1 (GLP-1) [49], highlights the “pseudo-organ” function, with unparalleled endocrine potential, of the gut microbiota. Unlike the endocrine organs of humans, which produce a limited number of hormones, the gut microbiota acts as a high-performance endocrine organ, capable of producing hundreds of humoral molecules recognized by human receptors, causing various biological effects [47]. The intestinal immune system is crucial for preserving the dynamic balance between the symbiotic microbiota and the host. A harmonious interplay among various local adaptive immune response elements, including secretory IgA and diverse regulatory T-cell responses, is essential for sustaining intestinal homeostasis. This interaction is necessary to confine microbes and microbial products within the gut lumen, ensuring the overall well-being of the intestinal environment [2,50,51].

In conclusion, emerging evidence supports the concept that the composition of the intestinal microbiota and its fundamental functions are related to physiological responses relevant to maintaining human health.

## 3. Gut Microbiota: Its Complexity and Dynamics

Dysbiosis, a term employed to delineate an imbalance in the composition and function of microbial ecology beyond its capacity to withstand and recover, is a crucial concept in the study of gut microbiota and immune system interactions. This imbalance can be affected by various factors, including environmental, dietary, xenobiotic, antibiotic, and genetic predisposition factors, depending on the specific context [42,52,53]. The ageing process itself can progressively damage the morphology and function of the intestinal microbiota, leading to a decrease in diversity and dynamics, an increase in *Bacteroidetes* and *Proteobacteria* spp., and a decrease in bifidobacteria, as observed in the ELDERMET study [15].

The absence or reduced abundance of essential components, alterations in metabolic processes, a decrease in alpha diversity (beneficial commensal bacteria), or an increase in pathogenic bacteria can all lead to the disruption of microbial homeostasis, resulting in dysbiosis in the ecosystem, and altering the microbial landscape. There are two categories of dysbiosis:

- Taxonomic dysbiosis: Taxonomic dysbiosis refers to an imbalance in the species composition of microorganisms within the environment. This imbalance may lead to a decrease in alpha diversity (beneficial commensal bacteria) or an increase in pathogens. We can observe the decline in microbial diversity at various taxonomic levels, including phylum, class, genus, and species. For example, in obesity-related diabetes, Firmicutes increases compared to Bacteroidetes, another prominent phylum [22]. *Firmicutes* possess genomes that metabolise food components more efficiently, increasing the risk of obesity.
- Functional dysbiosis: Researchers observe differences in the microbial genomic repertoires or microbial metabolites found in the gut or blood between healthy and ill individuals without detecting taxonomic alterations in the microbiota. For example, researchers associate alterations in the gut microbiota in irritable bowel syndrome (IBS) with the prevalence of NOD2 and CARD9 alleles [54].

Research has consistently shown a link between disease onset and dysbiosis in the gut microbiota. A shift in the gut microbiome can disrupt microbial symbiosis, influencing the immune system’s response. In the case of dysbiosis, new bacterial colonisation or a failure in everyday immunological protection can stimulate a distinct immune response [55]. This immune response can have significant health implications.

Environmental factors, such as nutritional changes, antibiotics, intestinal pH, xenobiotics, radio/chemotherapy, psychological and physical stress, iron intake, altered intestinal wall, the host’s genetics, extra-intestinal non-communicable diseases [56,57], and inflammatory bowel diseases, disrupt symbiosis. These factors alone can alter the intestinal milieu, potentially leading to the growth of pathobionts and their metabolites, which reach the bloodstream [58].

In healthy individuals, the intestinal barrier generally prevents the passage of microbes and substances from the intestinal lumen into the bloodstream. The maintenance of epithelial cell integrity is contingent upon forming tight junction complexes that act as a protective barrier against translocation along paracellular pathways [59]. These junctions consist of adhesive protein species, including occludins and claudins, serving as significant sealing proteins that prevent the diffusion of solutes and fluids. Proteins from the cytosolic of the zonula occludens (ZO) protein family and the peri-junctional ring of actin and myosin further regulate paracellular permeability. Notably, tight junctions exhibit adaptability, allowing them to adjust their tightness in response to physiological requirements [59]. They serve as a highly efficient barrier, safeguarding against the passage of microbes, lipopolysaccharides (LPS), toxic by-products of bacterial fermentation, digestive enzymes, and other harmful substances that may pose a risk of translocating from the gastrointestinal tract lumen to the internal milieu [46].

The intestinal immune system maintains a dynamic equilibrium between the symbiotic microbiota and the host. A well-balanced interaction among various components of local adaptive immune responses, including secretory IgA and diverse regulatory T-cell responses, is imperative for sustaining intestinal homeostasis [50]. This interaction contains microbes and microbial products within the gut lumen, as the literature emphasises.

The prevailing theory suggests that compromised gut barrier function may lead to the translocation of microbes into the bloodstream, sporadically resulting in systemic inflammation with significant clinical consequences (metabolic syndrome, reduced physical function, and mortality) [56]. This final theory challenges the reassuring notion of potential recovery from illness to health because the vicious cycle may facilitate crucial shifts and establish a permanently altered symbiosis, a feature possibly shared by most CD patients. These implications have substantial importance for innovation potential in the areas of prevention, diagnosis, and prediction [60], and they may pave the way for the development of targeted therapies addressing triggers, low microbiota richness, intestinal permeability, inflammation, and oxidative stress that permanently alter symbiosis following the critical transition.

## 4. The Role of Bacterial Metabolites as Pathophysiological Biomarkers

Increasing evidence highlights the significance of bacterial metabolites as pathophysiological indicators for chronic diseases, offering diagnostic and prognostic applications [61]. For instance, the plasma concentrations of trimethylamine N-oxide (TMAO), originating from choline and carnitine, demonstrate associations with diverse diseases [62,63]. Similarly, compounds like indoxyl sulphate (resulting from tryptophan breakdown) and p-cresyl sulphate (derived from tyrosine and phenylalanine), along with their metabolites [64], play roles in the advancement of kidney and vascular diseases. Regular dietary patterns play a significant role in moulding the composition of the intestinal microbiota and influencing the microbial metabolites that affect host metabolism (Table 2). For instance, researchers have associated following the Mediterranean diet (MD) with markedly elevated levels of total short-chain fatty acids (SCFAs), crucial metabolites produced by the gut microbiota that play a role in modulating immune-endocrine processes [65].

### 4.1. Short Chain Faty Acid (SFCA)

*Firmicutes* bacteria produce Short-Chain Fatty Acids (SCFAs) as products of fermentation and hydrolysis of dietary polysaccharides. After enterocytes absorb it, butyrate commonly serves as an energy source for the intestinal epithelium. Simultaneously, acetate and propionate enter the systemic bloodstream and reach distant tissues, contributing to lipid synthesis and glucose production [72].

SCFAs also function as signalling molecules, contributing to beneficial metabolic effects. These effects include the regulation of intestinal transit time [72], the modulation of food intake [73,74], an elevation in the intestinal uptake of monosaccharides (by stimulating the expression of sodium/glucose transporter-1), enhancing lipogenesis by inhibiting the inhibitor of lipoprotein lipase in the small intestine. This inhibition prevents the release of fatty acids from triglycerides and promotes the cellular uptake of triglycerides, stimulating their storage in depot organs [75].

SCFAs, particularly butyrate and propionate, have specific actions on colonic L cells. They do this by activating the G protein-coupled receptors GPR43 (Free Fatty Acid Receptor 2 or FFA2) and GPR41 (FFA3 or Free Fatty Acid Receptor 3). The activation of these receptors initiates a sequence of events that influence our appetite control. For instance, the activation of GPR43 leads to the release of PYY, an anorexigenic peptide, which increases gut transit rate and satiety. Simultaneously, the activation of GPR41 reduces inflammation, stimulates the release of glucagon-like peptides 1 and 2 (GLP1 and GLP2), and boosts levels of peripheral hormones such as leptin, insulin and ghrelin. These actions collectively influence appetite control [73].

### 4.2. Bile Acids

The gut microbiota potentially influences the regulation of cholesterol metabolism in the liver [76,77], and this affects the modification of bile acids that can impact systemic cholesterol levels [78]. Bile acids, primarily formed by the rate-limiting enzyme cholesterol 7-alpha-hydroxylase (CYP7A1) [79], represent key metabolites of liver cholesterol involved in the absorption of fats, nutrients, and lipophilic vitamins [80]. Additionally, they play a pivotal role in regulating lipids, glucose, and energy metabolism [81]. After conjugation with amino acids like taurine or glycine, enterocytes [80] absorb primary bile acids to form bile salts. Within the gut, the gut microbiota and bile salt hydrolase (BSH) deconjugate primary bile acids such as cholic acid (CA) and chenodeoxycholic acid (CDCA) to generate secondary bile acids, including ursodeoxycholic acid (UDCA), deoxycholic acid (DCA), and lithocholic acid (LCA) [80,81]. While most conjugated and unconjugated bile acids in the lumen are reabsorbed (95%) and transported back to the liver, UDCA and LCA are predominantly excreted in faeces.

Notably, signalling molecules like bile acids in the gut can activate the membrane G protein-coupled bile acid receptor Gpbar-1 (also known as TGR5) and the nuclear receptor farnesoid X receptor (FXR) [80,81]. This activation allows bile acids to downregulate bile acid synthesis [82], potentially leading to elevated cholesterol levels and contributing to CAD development. The sequence in which bile acids activate FXR is CDCA > DCA > LCA > CA. FXR induction can stimulate fibroblast growth factor 19 (FGF19), which, in turn, activates fibroblast growth factor receptor 4 (FGFR4) and suppresses CYP7A1, resulting in the downregulation of bile acid synthesis [83]. FXR can also decrease bile acid uptake into hepatocytes and increase biliary secretion of bile acids by upregulating the expression of ATP-binding cassette subfamily B member 11 (ABCB11). The division of primary and secondary bile acids may be involved in hypercholesterolemia and the development of coronary artery disease (CAD). Importantly, bile acids also play a significant role in cardiovascular function by modulating heart rate through the regulation of channel conductance and calcium dynamics in sinoatrial and ventricular cardiomyocytes and influencing vascular tone [84].

Moreover, an imbalance in gut microbiota modulation of bile acid ratios, particularly in an unhealthy state, could lead to decreased secondary bile acids, thereby increasing primary bile acids like CDCA. This imbalance underscores the need for further investigation into the gut microbiota and the underlying mechanisms involved—a crucial area for future research.

### 4.3. Lipopolysaccharides (LPS)

As previously noted, a complex network of tight junctions upholds the intestinal epithelial barrier, a crucial component of good health. This barrier’s primary function is to prevent the passage of antigens or microbe-derived endotoxins. However, certain pathological conditions can disrupt the balance of the gut microbiota, leading to a compromised intestinal barrier. In these instances, microbial metabolites [85] can breach the barrier, entering the bloodstream and initiating systemic pro-inflammatory signalling. This cascade of events can then trigger metabolic abnormalities in distant tissues, such as hyperglycemia, non-alcoholic fatty liver disease (NAFLD) and peripheral insulin resistance [67,86].

Metabolic endotoxemia, a condition that arises from the transport of pro-inflammatory molecules derived from microbes like LPS, flagellins, and peptidoglycans to the bloodstream, is a significant health concern. It contributes to the development or worsening of a wide range of human pathologies, spanning from intestinal conditions such as colon cancer and inflammatory bowel diseases to neurological disorders like Parkinson’s disease and autism. Moreover, it is associated with metabolic syndrome (MetS), obesity, transplant rejection, multiple organ failure, autoimmunity, traumatic brain injuries, depression, chronic fatigue, and HIV disease [87,88]. This broad spectrum of associated pathologies underscores the importance of understanding and addressing metabolic endotoxemia.

Researchers have meticulously documented the impact of LPS on insulin sensitivity in both in vitro and in vivo settings involving mouse models and human subjects. These studies have revealed that LPS promotes the differentiation of pre-adipocytes in culture, utilizing Janus kinase/signal transmitters and initiators of transcription (JAK/STAT) signalling and AMPK-regulated cytosolic phospholipase A2 (cPLA2) expression [89]. Human studies have further shown that exposure to *E. coli* LPS (3 ng/kg) induces systemic insulin resistance and inflammation related to adipose tissue [90]. Intravenous administration of a low dose (0.6 ng/kg) of LPS triggers a rapid, transient surge in plasma interleukin (IL)-6 (25-fold) and tumour necrosis factor α (TNFα; 100-fold), followed by a modest increase in the expression of pro-inflammatory cytokines (e.g., IL-6, TNFα, monocyte chemoattractant protein-1 (MCP-1), suppressor of cytokine signalling 1 and 3 (SOCS1 and SOCS3)) in adipose tissue [91]. Molecular studies in vitro demonstrate that LPS impairs insulin sensitivity by activating Toll-like receptors (TLRs). Specifically, LPS binds to LPS-binding protein, activates the CD14 receptor, and transfers TLR4 to the plasma membrane of macrophages [90]. This detailed understanding of the mechanisms involved in LPS’s effects on insulin sensitivity is crucial for further research and potential interventions.

In macrophages and dendritic cells, LPS play a regulatory role in nucleotide oligomerization domain (NOD)-like receptors, thereby initiating the activation of pro-inflammatory transcription factors such as interferon regulatory factors (IRFs), nuclear factor-κB (NF-κB) and activator protein-1 (AP-1). Various components of the inflammasome, such as caspase-1 and apoptosis-associated speck-like protein containing a caspase induction domain (ASC), are downstream targets of these events [89,92]. These orchestrated processes collectively contribute to the regulation of glucose and lipid homeostasis. Additionally, exploring the correlation between inflammatory markers and LPS serum levels in diabetic subjects would be an intriguing avenue for further research.

### 4.4. Uremic Toxins

The role of toxins generated by intestinal microbial metabolism is progressively acknowledged [93]. It is crucial to understand that intestinal bacteria degrade approximately 10 g of proteins in the colon daily. This process converts them into metabolites such as ammonia, amines, thiols, phenols, and indoles. These fermentation products in the colon are excreted in faeces, although a portion is absorbed and eliminated by the kidneys [48]. Among the uremic toxins derived from intestinal microflora are phenols and indoles: p-cresol and indoxyl sulphate. Notable among phenols are p-cresol, p-cresyl sulphate (PCS), p-cresyl glucuronide, phenylacetic acid, phenyl sulphate, and phenol [94]. P-cresol/p-cresyl sulphate is the product of phenylalanine and tyrosine metabolism by intestinal anaerobic bacteria. The intestinal wall converts p-cresol into PCS, which the liver metabolizes into p-cresyl glucuronide. PCS is the primary circulating metabolite of p-cresol [95]. Primarily originating from dietary intake, phenol is the result of tyrosine catabolism by the gut microbiota, as well as tobacco consumption.

Polyamines are organic cations that include cadaverine, spermine, spermidine, and putrescine. They originate from the intestine’s L-arginine, L-ornithine, or lysine decarboxylation. In CKD, putrescine, spermidine, and spermine are increased in serum.

Studies have demonstrated that these molecules interact with insulin and lipoproteins, contributing to hypertriglyceridemia, atherosclerosis, and increased CKD comorbidity.

### 4.5. Trimethylamine-N-Oxide (TMAO)

The clinically most relevant amine with high therapeutic and diagnostic potential is TMAO. It is crucial to note that researchers have identified it as a biomarker indicating the likelihood of significant adverse cardiovascular and cerebrovascular events, such as myocardial infarction and stroke. Elevated plasma concentrations of TMAO are connected, leading to the build-up of fatty accumulation in blood vessels: fatty liver, visceral obesity, and atherosclerosis. Elevated plasma concentrations of TMAO have been correlated with fatty deposits accumulated in blood vessels, fatty liver, visceral obesity, and atherosclerosis [96,97,98,99]. Choline precursors [100], phosphatidylcholine and carnitine, found in foods such as eggs, liver, red meat, and fish, are the primary dietary source of choline, a semi-essential nutrient belonging to the B-complex vitamin family [65]. Choline significantly impacts lipid metabolism and contributes to the synthesis of acetylcholine, homocysteine, and methionine [101].

Gut microbiota, predominantly *Enterobacteriaceae*, can metabolize choline, producing trimethylamine (TMA), dimethylamine (DMA), and monomethylamine (MMA). The microbial metabolism of TMA-containing nutrient precursors begins with specific microbial TMA lyases that generate TMA as a product. The major microbial choline TMA lyase is thought to encode the microbial cut C/D genes (cut gene cluster genes C [catalytic] and D) [102]. Microbes then produce TMA, which is transported to the liver via the portal vein and readily metabolized by host hepatic FMOs (flavin monooxygenases, mainly FMO376) into TMAO [103]. Consequently, dietary choline intake may lead to the generation of nitrosamine precursors with carcinogenic potential.

A meta-analysis study [104] demonstrated a positive dose-dependent association between brain/cardiovascular events, mortality, and circulating TMAO levels. A study of 330 adults with metabolic syndrome (MetS) found that high body mass index (BMI), visceral adiposity, and fatty liver index elevate circulating TMAO levels. The relationship between TMAO levels and MetS markers, such as blood pressure, serum glucose, obesity, serum lipids, and insulin resistance-related indices, was positive in 1081 subjects [61]. The METabolic Syndrome In Men (METSIM) study confirmed that the gut microbiota composition, which created high-level metabolites like TMAO, is *Bacteroidaceae*, *Ruminococcaceae*, *Lactinospiraceae*, and primarily *Peptococcaceae* and *Provotella* as the most dominant species. Plasma levels of TMAO are inversely associated with *Faecalibacterium parasitizing*.

**Figure 1:**
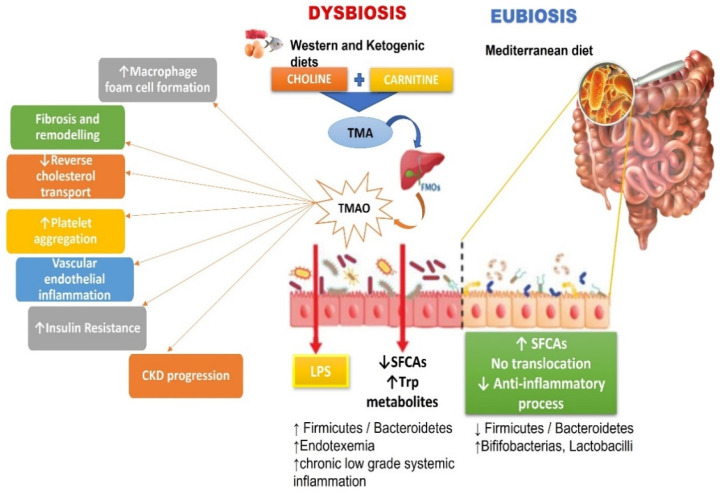
Gut microbiota dysbiosis: leads to an elevation in intestinal permeability, resulting in the translocation of lipopolysaccharide (LPS) and tryptophan-derived metabolites. This leads to subsequent metabolic endotoxemia and chronic low-grade systemic inflammation. The effects of trimethylamine-N-oxide (TMAO) are associated with several significant events that contribute to the onset and progression of chronic diseases. (Arrows indicating ↑ increase concentrations and ↓ decrease concentrations).

Moreover, TMAO may contribute to dyslipidaemia by regulating hepatic lipogenesis and gluconeogenesis [61], influencing macrophage scavenger receptors [97], and simultaneously downregulating cholesterol and bile acid metabolism [105] (Figure 1). Additionally, it hinders macrophage reverse cholesterol transport [80], facilitates the movement of activated leukocytes to endothelial cells [23], activates NF-κB signalling, and enhances platelet activation, promoting a pro-thrombotic phenotype [96]. It also induces endothelial dysfunction by activating the NLRP3 inflammasome [106]. Furthermore, TMAO has implications for brain functions, inducing neuronal senescence, increasing oxidative stress, impairing mitochondrial function, inhibiting mTOR signalling, and upregulating the expression of macrophage scavenger receptors and CD68. These phenomena collectively contribute to brain ageing and cognitive impairment [63,99,106]. 

The findings from these studies suggest a promising avenue for prevention and treatment in clinical practice. Analysing TMAO levels in serum or cerebrospinal fluid could represent a novel tool, offering new possibilities for managing health conditions.

### 4.6. Tryptophan Metabolism (Trp)

Tryptophan (Trp) is an aromatic amino acid found in oats, bananas, peanuts, dried prunes, milk, tuna fish, cheese, bread, poultry, and chocolate. The gut microbiota can directly utilize Trp, accounting for approximately 4–6%, thereby limiting its bioavailability [107]. Indoles derived from the bacterial metabolism of Trp play a crucial role in modulating physiological and pathological pathways in the host, contributing to conditions like cardiovascular, metabolic, and brain disorders. For instance, *Clostridium sporogenes* produces indole propionic acid from dietary Trp, which is essential for maintaining the integrity of the intestinal barrier.

Two other Trp metabolites, indoxyl sulphate and p-cresyl sulphate, stimulate GLP-1 in L cells, leading to subsequent insulin secretion from pancreatic β cells [108]. These metabolites appear to be associated with chronic kidney disease and related risk factors, including cardiovascular disease (CVD), hypertension, diabetes, and hyperhomocysteinemia [109]. 

However, existing studies are sometimes limited and contentious [110], highlighting the need for further investigations to identify potential diagnostic markers for the future.

## 5. Chronic Diseases: Correlation with Gut Microbiota

Considering bacteria’s significant role in the crucial oxygenation events [111] during the origins of life and our ongoing co-evolution, our lives are inevitably intricately linked with our body’s microbiome. Conditions such as heart disease, type 2 diabetes and dyslipidaemias, currently experiencing increased prevalence, have roots, at least in part, in a dysfunctional relationship between our gut microbiota and ourselves [8,112,113]. It is reasonable to believe that significant alterations have occurred over time that could impact the gut microbiome, thus contributing to the rise in the prevalence of various illnesses. At least two modifications may have substantially influenced gut microbiome dysbiosis over a few generations. 

Mode of birth: the number of caesareans increased compared to natural births. Infants born vaginally in the first year of life have lower levels of *Enterococcus* and *Klebsiella* spp. and higher levels of *Bifidobacterium* spp. compared to caesarean-born babies [114]. 

A diet high in cereals, refined sugars, refined vegetable oils, and alcohol, coupled with a more sedentary lifestyle, accounts for 72.1% of the daily energy intake [52]. Gut microbiota homeostasis is significantly affected both short- and long-term by diet and xenobiotics [38]. *Prevotella copri* and *Xylanobacter* thrive in high-fibre diets, whereas *Proteobacteria* prefer to grow on high-sugar diets [115].

Furthermore, xenobiotics significantly impact homeostasis. Medications such as antibiotics, antimetabolites, calcium-channel blockers, antipsychotics, antidiabetic drugs, nonsteroidal anti-inflammatory drugs, antiseptics, proton pump inhibitors, and antivirals alter the composition of the gut microbiota [116]. Polypharmacy regimens, which involve the simultaneous use of multiple medications, exert a particularly pronounced influence on interactions between drugs and the microbiome and play a pivotal role in both therapeutic and adverse health outcomes [117]. This aspect of research is of utmost importance, as clinical studies have linked polypharmacy with bacterial dysbiosis, reduced microbial diversity, and alterations in specific taxa such as *Prevotella*, *Parabacteroides*, and *Helicobacter* [118]. While the effects of polypharmacy on the gut microbiome are still not fully understood, they are increasingly recognized in clinical research focusing on microbiome shifts. These findings, which underscore the urgent need for further exploration of drug–microbiome interactions, especially in the context of polypharmacy, to better understand their impact on health and inform more personalized therapeutic approaches, are of paramount importance and demand our immediate attention [119].

Ageing, a complex and progressive biological process, is a significant factor that increases the incidence of chronic diseases. It is characterized by distinct hallmarks that culminate in the development of frailty and cognitive decline diseases [120]. These transformations can bring about individuals more susceptible to age-related chronic diseases, establishing a bidirectional vicious cycle of declining health. A recent meta-analysis, a testament to the complexity of this field, examined over 2500 gut microbiome datasets from individuals aged 20–89 with various conditions [118], such as Inflammatory Bowel Disease (IBD), CVD, T2DM, intestinal polyps, and colorectal cancer (CRC). The findings revealed specific taxa that not only increased across multiple diseases but also correlated significantly with increasing frailty in the ELDERMET cohort. While it remains challenging to determine whether these gut microbiome alterations are a cause or consequence of disease, the analysis of metabolic capabilities and gene presence/absence suggested an enrichment of pathways linked to the production of certain metabolites associated with disease onset [121].

Among the identified metabolites were Trimethylamine (TMA), secondary bile acids, and p-Cresol. Notably, there was a decrease in taxa known to produce Short-Chain Fatty Acids (SCFAs), metabolites that have been inversely associated with various diseases and metabolic abnormalities. These include chronic inflammation, insulin resistance, cognitive decline, obesity, epigenetic dysregulation, and impaired barrier function. The onset of age-related frailty is associated with specific bacterial taxa that exhibit metabolic capabilities that potentially contribute to the susceptibility to multiple clinical disorders [8,122].

In summary, alterations in the gut microbiome are associated with a range of metabolic disorders, as suggested by numerous research studies and publications, including the Human Microbiome Project (HMP) [60] and the European MetaHit Project [123]. The following section delves into the diverse effects of the gut microbiota and its metabolites on chronic diseases. It explores the causes and mechanisms of disease development in conditions such as chronic kidney disease (CKD), cardiovascular disease (CVD), and type 2 diabetes mellitus (T2DM).

### 5.1. Gut Microbiota Dysbiosis in Type 2 Diabetes Mellitus

Type 2 diabetes mellitus (T2DM), akin to cardiovascular disease, cancer, and chronic respiratory disease, is recognised as a chronic and noncommunicable ailment contributing to 80% of premature deaths globally [124]. If current trends persist, experts estimate that 700 million people will have T2DM by the year 2045 despite the availability of various pharmacological interventions [125]. Diabetes occurs when elevated blood sugar levels result from diminished pancreatic insulin production or reduced insulin sensitivity in tissues that ordinarily react to insulin signalling [113]. Inadequately controlled diabetes and metabolic disorders associated with type 2 diabetes, such as hypertension, impaired lipid metabolism and oxidative stress [13], can lead to both macrovascular and microvascular complications. Common macrovascular complications involving large blood vessels encompass coronary heart disease, cerebrovascular disease, stroke, peripheral vascular disease, congestive heart failure, organ inflammation, weight gain, impaired lipid metabolism, peripheral vascular disease, and electrolyte imbalance [13]. Microvascular complications involving small blood vessels include diabetic nephropathy, diabetic neuropathy, and diabetic retinopathy. Researchers have also observed alterations in interconnected metabolic pathways associated with T2DM [126]. For instance, coronary heart disease resulting from impaired insulin metabolism can lead to dyslipidaemia, a risk factor for cardiovascular complications in diabetes [127]. Specific factors contributing to the evolution of diabetes complications comprise elevated chronic hyperglycaemia, decreased antioxidant status and reactive oxygen species (ROS) [128]. These complications not only lead to an overall decline in quality of life but also an increase in mortality rate.

Numerous studies have illustrated a significant correlation between alterations in the composition profile of gut microbiota and the development of diabetes. Specifically, disrupted *Bacteroidetes*/*Firmicutes* phylum eubiosis has been associated with heightened intestinal permeability [112], allowing bacterial by-products to infiltrate through a leaky gut barrier, triggering subsequent inflammatory responses characteristic of diabetes. Also, the reduced abundance of butyrate-producing bacteria and SCFAs, particularly butyrate, has been directly associated with type 2 diabetes mellitus (T2DM), owing to its connection with insulin sensitivity [129]. This relationship between SCFAs and insulin sensitivity is attributed to SCFAs’ unique ability to stimulate the secretion of GLP-1 by intestinal L-cells through specific G protein receptors (GPR41, GPR43). This stimulation significantly influences pancreatic function and insulin release and centrally regulates appetite [130,131]. Some research provides a deeper understanding of these mechanisms, shedding light on the potential for microbiome-based interventions in T2DM management. Conversely, certain bacteria have demonstrated a protective role by reducing the risk of diabetes development by decreasing proinflammatory markers and maintaining intestinal barrier integrity. For example, *Lactobacillus fermentum*, *L. plantarum*, *L. casei*, *Roseburia* intestinalis, *Bacteroides fragilis*, *and Akkermansia muciniphila* have all exhibited the capacity to enhance glucose metabolism insulin sensitivity and suppress proinflammatory cytokines [13].

Furthermore, in individuals with diabetes-associated gut dysbiosis, metformin [132] fosters butyrate and propionate production, enhancing a patient’s ability to catabolise amino acids [133]. These changes, combined with heightened levels of *Akkermansia* in the gut, may influence the effects of metformin on glucose metabolism. Metformin, a frequently prescribed medication for diabetes treatment, is known for its ability to curb liver glucose production, enhance insulin sensitivity, and boost muscle and liver glucose absorption [134]. The gut microbiome influences the effectiveness of metformin. Both animal and human studies have shown that metformin intake leads to an increase in A. muciniphila levels and various bacteria known for Short-Chain Fatty Acid (SCFA) production, such as *Blautia* and *Butyricicoccus* [135]. *A. muciniphila*, a key player in glycemic control, promotes ileal goblet cell growth, reduces gut permeability, lowers endotoxemia, and stimulates TLR signalling in mouse models. This suggests that the gut microbiota, particularly *A. muciniphila*, might play a crucial role in metformin’s efficacy and its gastrointestinal tolerance.

The SCFA butyrate supports energy metabolism in rodents by benefiting skeletal muscle, brown fat tissue, and pancreatic β-cells117. Additionally, propionate SCFA inhibits liver gluconeogenesis and curbs appetite and weight in rodent studies [136].

Gastrointestinal discomfort, including pain, bloating, and nausea, ranks among metformin’s most common side effects. However, a study involving 27 non-diabetic men found that specific genera (*Sutterella*, *Allisonella*, *Bacteroides*, and *Paraprevotella*) in stool samples before starting metformin correlated with subsequent gastrointestinal side effects [137]. This intriguing finding suggests that the gut microbiota might influence both metformin’s efficacy and its gastrointestinal tolerance. Therefore, the potential of stratifying patients based on microbiome profiles could be a significant step forward, helping to identify those likely to respond well and tolerate therapeutic doses and offering hope for more personalized diabetes management. Limited data support the role of the gut microbiome in other diabetes treatments, but a reduction in *Firmicutes* levels in mice treated with the GLP1 agonist liraglutide correlated with improved glycaemic control [138].

The metabolic factors associated with oxidative stress and chronic low-grade inflammation, linking gut microbiota dysbiosis and T2DM, appear to be the same factors shaping the onset and progression of diabetic complications [139,140]. For instance, diabetic nephropathy affects approximately 40% of individuals with poorly managed diabetes, leading to complications such as end-stage renal disease and cardiovascular issues. Hyperglycaemia-induced stress on the kidneys results in systemic inflammation, albuminuria, and proteinuria [67]. Various factors, including genetics, age, obesity, hypertension, and dyslipidaemia, contribute to the progression of diabetic nephropathy. Recent research has emphasised the role of gut microbiota dysbiosis occurrence and that progression induces alterations in the composition of the gut microbiota, particularly a reduction in beneficial bacteria such as *Prevotella* [141], Ruminococcaceae, *Roseburia*, *Faecalibacterium*, coupled with an elevation in the prevalence of *Parabacteroides*, *Enterococcus*, *Enterobacteriaceae*, and *Klebsiella* [22]. Another microvascular problem linked to T2DM is diabetic retinopathy, a severe complication that may result in blindness over time. Increased oxidative stress, inflammation, and gut microbiota dysbiosis are linked to diabetic retinopathy [139]. The microbiota composition varies between body compartments, with the ocular surface hosting *Proteobacteria* and *Actinobacteria*. Patients with diabetic retinopathy exhibit altered microbiota profiles, including decreased *Bacteroidetes* and *Actinobacteria* and increased levels of *Acidaminococcus*, *Escherichia*, and *Enterobacter*. TMAO is associated with the severity of diabetic retinopathy [142]. 

Additionally, changes in the gut microbiota, featuring increased *Firmicutes*, *Actinobacteria*, *Escherichia-Shigella*, *Lachnoclostridium*, *Blautia*, *Megasphaera*, and *Rumincoccus*, along with decreased *Bacteroidetes* and *Faecalibacterium*, are associated with diabetic neuropathy [143], a neurodegenerative disease causing peripheral nerve damage and symptoms like pain and numbness. Diabetic neuropathy is characterised by declined peripheral innervation, neuronal inflammation, demyelination, axonal atrophy, and reduced regenerative capacity. It affects around 50% of diabetic patients, resulting in complications such as cardiovascular damage, tachycardia, orthostatic hypotension, and hormonal imbalance. Factors such as oxidative stress, polyol pathway activation, inflammation, and insulin resistance contribute to its development [144].

Strategies to balance the gut microbiota, such as probiotics and symbiotics, demonstrate promise in modulating T2DM. The study showed a promising trend toward better glycaemic control with a combination synbiotic therapy (*A. muciniphila*, *Clostridium beijerinckii*, *Clostridium butyricum*, *Bifidobacterium infantis*, *Anaerobutyricum hallii*, and inulin) compared to a placebo. This encouraging result, although with a small sample size and short 12-week follow-up, leaves the potential long-term benefits for T2DM uncertain [145]. However, it does underscore the promise of further research and similar targeted microbiome approaches, which could pave the way for more precise diabetes management in the future.

### 5.2. Gut Microbiota Dysbiosis in Cardiovascular Diseases (CVD)

Cardiovascular disease (CVD), encompassing conditions like hypertension, atherosclerosis, cardiomyopathy, and heart failure, remains a significant cause of morbidity and mortality globally, imposing substantial health and economic burdens [146]. A plethora of evidence indicates that alterations in the gut microbiota composition can influence cardiovascular phenotypes [45].

Hypertension, recognized as the most prevalent risk factor associated with CVD, stands as a leading cause of disability and death in developed countries [147]. Individuals with hypertension exhibit lower gene richness and alpha diversity than healthy controls and demonstrate a higher percentage of bacteria from the *Prevotella* [148]. Researchers have observed strong correlations between hypertension and gut microbiota dysbiosis, suggesting a potential causal relationship between elevated blood pressure and changes in the gut microbiota. Changes in gut microbiota composition associated with hypertension accompany alterations in bacterial metabolic products [149], driven by an increased Firmicutes/Bacteroidetes ratio [49,150,151].

The gut microbiota may play a part in salt sensitivity associated with hypertension. In the study by Bier et al., a high salt diet (HSD) induced hypertension in rats, leading to increased levels of taxa from the *Erwinia* and the *Corynebacteriaceae* families. Conversely, *Anaerostipes* exhibited decreased abundance compared to the control group [152]. HSD specifically affects the rodent gut microbiota by decreasing the abundance of *Lactobacillus murinus* and inducing T helper 17 cells, resulting in salt-sensitive hypertension [153]. Daily *L. murinus* supplementation ameliorates these effects [154]. Several studies suggest antihypertensive medications may lower blood pressure by influencing the gut microbiota. Angiotensin II receptor blockers, such as candesartan, have been reported to normalize the F/B ratio, preserve *Lactobacillus* levels, and prevent gut microbial disruption [155,156].

Recent studies have revealed bacterial translocation from the gut to the heart [157] and the presence of gut bacterial DNA in plaques. Metagenomic analysis identified differences in the gut microbiome of atherosclerotic cardiovascular patients, showing elevated levels of *Streptococcus* and *Enterobacteriaceae* spp. [158]. Gut dysbiosis can contribute to atherosclerosis through metabolism-dependent pathways, with TMAO playing a significant role [148,159]. TMAO is associated with coronary plaque vulnerability, influencing inflammation and foam cell formation [104]. Changes in bacterial composition associated with dysbiosis can result in heightened intestinal permeability, leading to increased circulating LPS levels. The detected LPS can activate Toll-like receptor 4 (TLR4) [160], and subsequent signals are transduced by myeloid differentiation primary response 88 (MYD88), promoting inflammation and the formation of foam cells [161]. Additionally, TLR-sensed signals induce B2 cell activation in the spleen, altering IgG production and contributing to atherosclerosis development [162].

TMAO-dependent mechanisms involve the upregulation of macrophage scavenger receptors and CD36 expression, disrupting cholesterol metabolism in macrophages and fostering foam cell generation [163]. TMAO also hinders the hepatic bile acid synthetic rate-limiting enzymes Cyp7a1 and Cyp27a1, leading to reduced cholesterol elimination and reverse cholesterol transport (RCT) [164]. Moreover, TMAO induces vascular endothelial dysfunction through NF-κB and inflammasome activation, heightening the expression of vascular endothelial inflammation factors [165].

The correlation between TMAO levels and thrombotic events introduces the possibility of using TMAO as a therapeutic target and a diagnostic marker for subjects at risk of CVD-related consequences. The gut microbiome’s influence on the effectiveness and potential side effects of various cardiovascular disease (CVD) treatments is not just significant—it is crucial. For instance, *Eggerthella lenta* harbours cardiac glycoside reductase genes that deactivate digoxin, a crucial cardiac arrhythmia treatment drug inhibiting Na^+^/K^+^/ATPase in the heart muscle [166]. This inactivation likely results from the enzyme’s ability to process multiple substrates rather than evolving specifically due to digoxin exposure. Such instances underscore the gut microbiome’s chemical diversity and its profound impact on human drug metabolism [38]. Given digoxin’s narrow therapeutic range, identifying this bacterial metabolic pathway before treatment could enhance dosing accuracy and reduce adverse effects, a fact that underscores the importance of this research.

Statins, commonly used to treat CVD-related hyperlipidemia by inhibiting HMG-CoA reductase, show varied effectiveness. Response variability might stem from gut microbiome differences, as individuals with higher microbial diversity show more robust statin responses [167], as in certain animal models [168,169,170]. Notably, higher *Proteobacteria* levels correlate with reduced simvastatin efficacy [40], and different microbial compositions influence rosuvastatin’s effectiveness. Recent research has linked statin treatment with changes in gut microbiota, like the reduced prevalence of the obesity-associated *Bacteroides* 2 enterotype [171]. Whether this can guide treatment decisions and predict outcomes remains to be explored.

With the growing recognition of the gut microbiome’s role in CVD, numerous clinical trials are exploring the potential benefits of probiotics. For instance, studies are comparing rifaximin with the probiotic *Saccharomyces boulardii* [172] and investigating the impact of Lactobacillus acidophilus on heart failure-related inflammation. These trials are not only shedding light on microbiome changes post-treatment but also hinting at the promising potential of probiotics in CVD management.

Some findings suggest promising avenues for next-gen microbial treatments focusing on specific functions or barrier enhancement. Alternatively, targeting specific microbial pathways with small-molecule inhibitors, like those inhibiting TMA production [173,174], could enable more tailored interventions, particularly for patients with elevated TMAO levels [175].

In conclusion, comprehending the gut microbiome’s role in CVD pathogenesis and treatment opens up new horizons for disease identification, stratification, and management. This understanding could potentially revolutionize how we approach and manage CVD, offering a more personalized and effective treatment approach.

Further exploration of these mechanisms is needed to understand the role of gut microbiota in atherosclerosis and thrombosis.

### 5.3. Gut Microbiota Dysbiosis in Chronic Kidney Diseases (CKD)

Dysbiosis in the intestinal microbiota is a critical factor in the earliest stages of CKD. This dysbiosis affects the quantity and quality of the microbiota’s composition and alters the metabolites it produces. By influencing the metabolic, endocrine, or immune systems, these conditions can potentially initiate or exacerbate CKD, emphasizing the immediacy and importance of understanding and addressing this issue [46].

CKD is a noteworthy public health concern, affecting 6–10% of the adult population in various countries [176]. In CKD patients, dysbiosis leads to a decrease in *Akkermansia* levels, a key player in enhancing intestinal barrier function and mucus thickness [177]. This dysbiosis also contributes to hydrogen sulphide detoxification. *Proteobacteria* in the gut are implicated in triggering inflammation, disrupting mucosal permeability, and elevating the ratio of T helper 17 to regulatory T cells, promoting endotoxin translocation. Within the population affected by chronic kidney disease, there is an observed increase in *Bacteroidetes* and *Proteobacteria*, coupled with a decrease in *Lactobacillus* from the *Firmicutes* phylum, compared to their healthy counterparts [178]. Individuals with CKD exhibit elevated counts of *Proteobacteria (Escherichia* and *Shigella*). Meanwhile, there is a decrease in the abundance of *Roseburia*, *Faecalibacterium*, and *Prevotella* [179]. The reduction in these bacteria leads to decreased butyrate production, a compound known for its kidney-protective properties [180]. Butyrate inhibits histone deacetylases, mitigating fibrosis and improving acute kidney injury-induced damage [181]. Moreover, it exhibits anti-inflammatory properties as an agonist for ‘G protein-coupled receptors’, involved in regulating inflammation [182].

Disease conditions, such as decreased glomerular filtration rate in CKD, result in modifications to gut microflora through the accumulation of metabolites [183]. Changes in the gut microbiota under CKD conditions contribute to increased intestinal permeability, facilitating the rise of endotoxins like lipopolysaccharides (LPS) in the bloodstream [15]. This disruption of blood homeostasis can lead to atherosclerosis and an elevated risk of mortality among CKD patients [184]. The increase in uremic metabolites resulting from gut microflora contributes to an upsurge in inflammation, oxidative stress, and deimmunization [15,184,185].

The entry of urea into the gastrointestinal tract leads to hydrolysis into ammonia, increasing intestinal pH and causing inflammation and erosion of the intestinal wall. Bacteria such as *Alteromonadaceae* and *Clostridiaceae*, which produce urease enzymes, are more abundant in patients with end-stage renal disease. Ammonia affects gastric epithelial cells, reducing epithelial barrier reinforcement, disrupting tight junction proteins, and increasing intestinal permeability [46]. The reduction in butyrate levels in CKD diminishes mucin production and tight junction proteins, which have anti-inflammatory properties [133]. Additionally, macrophages in the intestine contribute to inflammation, potentially leading to endotoxemia and systemic inflammation. Disruption of tight junction complexes weakens the epithelial barrier, making cells more susceptible to stress, affecting cell polarity, and promoting pathogen growth. Fluid retention in CKD and circulatory stress induced by haemodialysis contribute to increased endotoxin translocation from the gut. Also, activation of the NF-kB pathway triggered by elevated levels of microbial metabolites, such as p-cresol, trimethylamine, and indole propionic acid, induces systemic inflammation [185,186].

A characteristic representative of water-soluble uremic toxins includes TMAO, which disrupts blood homeostasis in individuals with CKD, inducing platelet hyperactivity and lipid disorders [184]. It achieves this by promoting metabolic bacteraemia and endotoxemia. Simultaneously, TMAO diminishes the expression of angiopoietin-like protein 4, which inhibits lipoprotein lipase activity and stimulates lipolysis in white adipose tissue [187].

While the gut microbiota’s role in CKD progression has garnered attention in recent years, the potential of comprehensive systemic studies and gut microbiota corrective interventions to restore healthy gut conditions is a promising avenue for mitigating CKD progression.

## 6. Gut Microbiota: Opportunities and Challenges

Over the past ten years, a wealth of evidence from both animal and human studies has underscored a profound link between the gut microbiome and a range of chronic conditions. These include inflammatory autoimmune diseases, gastrointestinal inflammation-related conditions, and cardiometabolic disorders. Bacterial metabolites, particularly short-chain fatty acids (SCFAs), are now widely acknowledged as pivotal contributors to the gut microbiome’s influence on human health [14].

Bacteria that produce butyrate are linked to a reduced risk of inflammatory autoimmune diseases, cardiometabolic conditions, and irritable bowel syndrome. While several therapeutic approaches can target the gut microbiome, dietary modifications are a straightforward, non-invasive, and immediate method to influence its composition and function [188]. Recent randomized experiments have consistently demonstrated that specific dietary changes lead to predictable responses in the gut microbiome’s composition and function.

By incorporating dietary fibre and unsaturated fats, either individually or as part of a balanced diet like the Mediterranean diet [65,189], we can foster a higher abundance of butyrate-producing bacteria. These bacteria, along with the SCFAs generated, significantly impact advancing health benefits. However, it is important to note that the effects of dietary modifications on the gut microbiome are still being studied, and the optimal dietary strategies for specific health conditions are not yet fully understood. Importantly, different dietary fibres can induce specific shifts in bacterial populations and SCFA production. The possibility of developing dietary interventions customized to enhance specific bacterial metabolites, thereby ameliorating cardiometabolic and inflammatory health outcomes, appears within reach in the near future.

The intricate interplay between enteric microbial symbionts and host immunity has sparked a myriad of strategies to manipulate the gut microbiota for managing and preventing chronic diseases. Clinical approaches involve methods such as antibiotics, antifungal agents, dietary modulation, and live microbe supplementation. Proposed therapeutic strategies, including prebiotics, probiotics, postbiotics, and TMAO-synthesis inhibitors, aim to target the gut microbiome, with faecal microbial transplantation (FMT) [190] emerging as a promising intervention in various conditions [191].

While animal models successfully treat inflammatory conditions through gut microbiota manipulation, human trials present less conclusive data. Findings underscore the importance of considering the existing structure of the resident gut microbiota in microbial intervention-based clinical trials [1], potentially explaining disparities between animal models and human trials. Live microbial supplementation studies have yielded encouraging results, emphasizing the need to give attention to microbial strain selection and tailoring treatment to the recipient’s endogenous gut microbiome.

Ongoing studies strive to unravel the basis of microbe–microbe interactions to identify specific gut microbiomes responding more readily to targeted microbial interventions. Tailored interventions, considering the microbial individuality of recipients, are not just crucial but pivotal for preventing or managing chronic diseases [6]. This shift toward personalized multispecies microbial consortia sourced from healthy human enteric ecosystems is expected to replace traditionally used probiotic strains.

In addition to gut microbiota interventions, certain plant-specific ingredients, such as curcumin, resveratrol, and genistein, profoundly affect specific genes. Curcumin [192], for instance, inhibits various cell-signalling pathways akin to the potential effects of supplemented probiotics. Studies on gene expression after exposure to specific lactic acid bacteria highlight strain-specific and diverse expression profiles induced by different probiotics. These effects resemble responses to various foods, especially plant ingredients, and parallel observations seen with certain pharmaceuticals. The responsiveness to various lactic acid bacteria (LAB) [193] is influenced not only by genetic background and existing microbiota but also by lifestyle and diet, which may explain differences in individual responses observed in studies and variations in outcomes seen in clinical probiotic studies, particularly in critically ill patients.

The discussion on the role of prebiotics and probiotics in promoting gut health and managing metabolic disorders underscores the significance of these interventions in the broader context of personalized microbial therapeutics [194]. The complex interplay between enteric microbial symbionts and host immunity, as explored in the previous section, creates a framework for understanding the potential of prebiotics and probiotics in preventing and managing chronic diseases.

As the text delves into the specifics of prebiotics, emphasizing their selective fermentation and ability to induce changes in gut microorganisms, the connection with the broader theme of gut microbiota manipulation becomes evident. Oligosaccharides like fructo-oligosaccharides (FOS) and galacto-oligosaccharides (GOS) are highlighted for their influence on beneficial bacteria, such as *Bifidobacterium* and *Lactobacillus*, aligning with the broader strategy of manipulating gut microbiota discussed earlier [164].

Symbiotic, a term coined by the pioneering work of Gibson and Roberfroid in 1995 [195], combines probiotics and prebiotics to modulate the gut microbiome synergistically for therapeutic benefits. This innovative concept enhances the survival of live microbial supplements in the gut while promoting beneficial bacteria growth. Prebiotics like fructooligosaccharides FOS stimulate beneficial bacteria such as Bifidobacteria and Lactobacilli while inhibiting harmful strains like Bacteroides and Clostridia [196]. Excitingly, studies show symbiotics can significantly reduce inflammation, improve gastrointestinal symptoms, and even slow chronic kidney disease progression. Common combinations include Bifidobacteria or Lactobacilli with prebiotics. Symbiotics offer a potent means to target gut dysbiosis-related diseases and promote overall health [197]. Further research is needed to explore their full potential in preventive and therapeutic medicine. The introduction of next-generation probiotics, with a focus on specific commensal species like *Akkermansia muciniphila* [198], seamlessly extends the discussion on gut microbiota manipulation and its potential therapeutic implications. The association of *A. muciniphila* with health benefits and its potential as a therapeutic agent for metabolic syndrome (MetS) further aligns with the overarching theme of personalized microbial treatment. This exciting development paves the way for a new era in microbiome-based therapeutics.

The exploration of fermented products like yoghurt and kefir, with their positive effects on various health aspects, connects with the broader context of dietary modifications and nutritional support discussed earlier [188]. The cautionary note on interpreting study results due to the heterogeneity of probiotic strains echoes the need for tailored interventions and personalized approaches.

In summary, the integrated approach to microbiome-based therapeutics, combining insights from gut microbiota manipulation, plant-specific ingredients, and the potential of prebiotics and probiotics, underscores the complexity of microbial interactions. However, the ongoing research and emphasis on personalized interventions are crucial for advancing our understanding and realizing the full potential of these interventions in promoting gut health and managing metabolic disorders that genuinely drive our pursuit of knowledge in this field.

## 7. Conclusions

In conclusion, the intricate interplay between visible and invisible cellular components of the human body underscores the multifaceted nature of human physiology. While visible organs are readily evident and subject to medical intervention for maintenance and repair, the microbiome, though invisible, equally plays a pivotal role in maintaining health and well-being. Disruptions or dysbiosis in the microbiome can compromise its delicate balance and functionality, arising from various factors, including diet, medications, and lifestyle choices. Similar to visible organs, medications, surgical procedures, or lifestyle modifications are required to restore microbial harmony and promote health. The gut microbiome’s role extends beyond being the direct cause of chronic diseases; it often contributes to diseases within a system’s biology involving host genetics, physiological responses, and environmental factors. Quantifying the microbiome’s relative contribution to chronic diseases compared to other variables remains challenging due to the intricate relationships between host, environment, and microbiome. While the field has seen significant advancements in understanding the microbiome’s role in health and CD, challenges such as lack of validation of microbiome-based markers, standardization in data processing and analysis, and individualized understanding of microbiome-related mechanisms persist. However, the rapid progress in the field over the past decade instils optimism that the microbiome will soon become an integral part of clinical practice, offering new avenues for personalized treatment strategies. Moving forward, a multidimensional approach incorporating host and microbial multi-omics, exposome, and longitudinal data analysis is essential. This approach, along with innovative in vitro and ex vivo methods, will aid in identifying novel factors responsible for treatment response variability and chronic disease states. As the field continues to evolve, it is crucial to proceed with caution and focus on addressing the existing challenges to effectively translate microbiome knowledge into clinical applications, benefiting patients through personalized treatment strategies.

## Figures and Tables

**Table 1:** Roles of gut microbes from the most prevalent phyla.

Phyla	Family	Effects
Firmicutes	*Ruminococcus, Clostridium*, *Lactobacillus*, *Anaerostipes*, *Eubacterium*, *Faecalibacterium*, and *Roseburia*	- Hydrolyse complex carbohydrates in the gastrointestinal tract that are resistant to digestion by the body’s intrinsic enzymes [11] - SCFA production [27] - Synthesis of antimicrobial, anti-carcinogenic, and anti-inflammatory molecules and peptides [22]
Bacteroidetes	*Bacteroides*, *Prevotella*, *Clostridiales* and *Xylanibacter*	- Metabolize complex carbohydrates through fermentation, generating volatile fatty acids that serve as an energy source for the host [28] - Stimulate the proliferation of mutualistic bacteria with increased dietary fibre intake [29] - Induce metabolic alterations in the microbiota, resulting in decreased IL-18 production, mucosal inflammation, and the potential development of systemic autoimmunity [30]
Actinobacteria	Bifidobacteria	- Maintaining gut homeostasis [16] - Probiotic [31]
Proteobacteria	*Escherichia coli* and *Salmonella*	- An imbalanced rise results in a compromised gut microbiota and inflammation [27]
Verrucomicrobia	*Akkermansia muciniphila*	- Enhances gut barrier function and exhibits anti-inflammatory properties [19]

**Table 2:** Gut microbiota metabolites, dietary factors, and host responses.

Family	Diet	Metabolites	Effects
Prevotellaceae [66]	Mediterranean diet	SCFAs	➢ Source of energy ➢ Cell communication molecules ➢ Control of gastrointestinal transit duration ➢ Regulation of host appetite and food consumption.
Enterobacteriaceae [67,68,69]	Western diet	LPS	➢ Metabolic endotoxemia ➢ Pro-inflammatory signalling.
Bacteoridaceae Enterobacteriaceae, Lachnospiraceae Ruminococcaceae [55,62,63]	Dietary source of choline	TMAO	➢ Risk factors for insulin resistance, obesity, hypertension ➢ Chronic kidney diseases ➢ Cardiovascular, and cerebrovascular events.
Clostridiaceae [70,71]	Very low carbohydrate ketogenic diet.	Indoxyl sulphate, p-cresyl sulphate	➢ Chronic kidney disease ➢ Cardiovascular, metabolic ➢ Brain disorders.
